# Follow the “DOTs”: Vδ1^+^ γδ T cells as effectors of cancer immunotherapy

**DOI:** 10.1084/jem.20252289

**Published:** 2026-02-11

**Authors:** Bruno Silva-Santos, Sofia Mensurado, Rafael Blanco-Domínguez, Adrian C. Hayday

**Affiliations:** 1 Gulbenkian Institute for Molecular Medicine, Lisbon, Portugal; 2 Faculdade de Medicina da Universidade de Lisboa, Lisbon, Portugal; 3 King’s College London, London, United Kingdom; 4 The Francis Crick Institute, London, United Kingdom

## Abstract

Cancer immunotherapy has mostly relied on conventional T cells to achieve success in a limited set of tumour types. A promising avenue to expand the repertoire of cancers effectively treated through immune intervention is to mobilize other anti-tumour effectors, such as γδ T cells. Among these, the Vδ1^+^ subset commonly predominates within peripheral tissues and within tumours, typically associating with good prognosis. In this *Found in Translation*, we discuss how to leverage the biological properties of Vδ1^+^ γδ T cells for cancer immunotherapy, with special focus on the Delta One T (DOT) cell approach.

## Vδ1^+^ γδ T cells and the “DOT” cell product

γδ T cells are defined by T cell receptors (TCRs) produced following somatic recombination of genes encoding TCRγ and TCRδ chains. Like antibodies, TCRγδ complexes are rarely restricted by major histocompatibility complex (MHC) / human leukocyte antigen (HLA) and do not recognise processed peptides, unlike conventional □□ TCRs (Hayday et al., 2024). While the identity of antigens for most of the TCRγδ repertoire is unelucidated, the dominant γδ T cell population in human peripheral blood, Vγ9Vδ2 T cells, engage a complex of butyrophilins (BTNs) 2A1, 3A1, and (most likely) BTN3A2 where BTN2A1 interacts directly (in the extracellular space) with the Vγ9 TCR chain, and BTN3A1 binds intracellularly to prenyl pyrophosphates (“phosphoantigens”) that accumulate in tumour or infected cells (Mohammed et al., 2025).

Although Vγ9Vδ2 T cells clearly display anti-tumour effector functions, namely cytoxicity and production of type 1 cytokines, e.g., interferon (IFN)-γ or tumour necrosis factor (TNF), their clinical translation has been limited by poor expansion in vivo and frequent propensity to exhaustion (Hayday et al., 2024). Conversely, we and others have found that their Vδ1^+^ γδ T cell counterparts, which are typically rare in the blood but which predominate in peripheral tissues and within tumours, are more resistant to exhaustion, retaining substantial effector responses to TCR signalling (Correia et al., 2011; Davies et al., 2024). Moreover, tumour-infiltrating Vδ1^+^ but not Vδ2^+^ γδ T cells associated with good prognosis in breast (Wu et al., 2019) and lung (Wu et al., 2022) carcinoma patients; and expanded upon PD-1 immune checkpoint blockade (ICB) in colorectal cancer patients with HLA class I defects (de Vries et al., 2023). Clearly, these properties make Vδ1^+^ γδ T cells attractive effectors of cancer immunotherapy.

Aiming to develop a Vδ1^+^ γδ T cell-based therapeutic product, we tested thousands of conditions with TCR agonists and cytokines to arrive at the “Delta One T (DOT) cell” protocol that in 2-3 weeks yields >1,000-fold expansions of blood-derived Vδ1^+^ γδ T cells, constituting >70% of the final γδ T cell product (Almeida et al., 2016). Over the past decade, we have tested the therapeutic potential of DOT cells in multiple preclinical models of haematological and solid cancers, leading to a first-in-human clinical trial in acute myeloid leukaemia (AML) (NCT05886491).

## DOT cells in haematological malignancies

Haematological malignancies provided the first conceptual and experimental framework to test the therapeutic potentials of DOT cells. Given that circulating Vδ1^+^ γδ T cells were increased in a subset of patients with B-cell chronic lymphocytic leukaemia (CLL) and associated with favourable clinical outcomes (Poggi et al., 2004), DOT cells were tested in preclinical models of this disease. We showed that DOT cells efficiently recognize and target CLL cell lines and primary autologous and allogeneic patient samples (Correia et al., 2011). Moreover, DOT-cell cytotoxicity shows an attractive therapeutic window in being largely selective for malignant versus healthy lymphocytes, consistent with tumour-specific rather than lineage-restricted recognition. In xenograft CLL models, adoptively transferred DOT cells infiltrated tumours and other organs, including spleen, bone marrow, and liver, where they sustained a stable type 1 effector profile characterized by IFN-γ and TNF without evidence of functional exhaustion or diversion toward IL-17 production (Almeida et al., 2016). Importantly, DOT-cell treatment improved mouse survival and limited systemic CLL dissemination, a critical feature in a disease defined by widespread tissue involvement (Almeida et al., 2016).

We next evaluated the therapeutic potential of DOT cells in AML, which is characterised by frequent chemoresistance and very poor survival rates, particularly, although not exclusively, among elderly patients. DOT cells displayed potent cytotoxicity against primary AML samples and a broad panel of AML cell lines, including some extensively treated and resistant to standard chemotherapy (Di Lorenzo et al., 2019). Clonal tracking further showed that, unlike chemotherapy, DOT-cell treatment did not select for resistant leukaemic subclones, but instead preserved the clonal architecture of AML cell populations, enabling repeated tumour elimination without evidence of immune escape. Adoptive transfer of DOT cells in cell line-based or patient-derived xenograft models substantially reduced leukaemic burden in the blood and target organs, including bone marrow and liver, and significantly prolonged mouse survival without detectable toxicity (Di Lorenzo et al., 2019; Sánchez Martínez et al., 2022).

**Figure 1:**
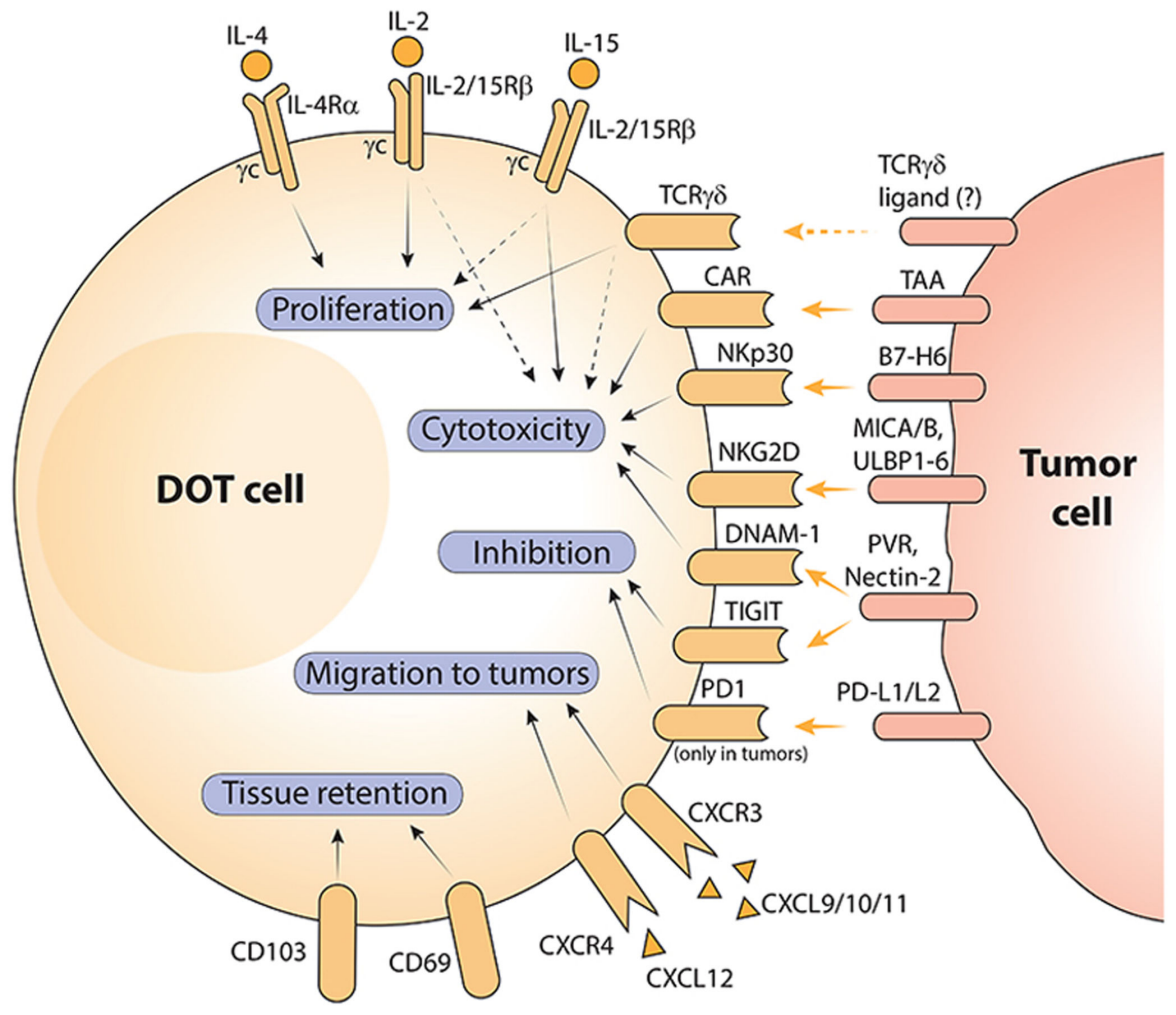
Phenotype and regulatory mechanisms of Delta One T (DOT) cells. DOT cells are in vitro-expanded γδ T cells that mostly (> 70%) express a Vδ1^+^ T cell receptor (TCR), which controls their activation, proliferation and differentiation during the 2-3 week protocol (Almeida et al., 2016). The cytokines IL-2 and IL-4 provide important signals for DOT-cell proliferation, whereas IL-15 drives their cytotoxic effector phenotype. During in vitro expansion, strong TCR stimulation in the presence of IL-15 upregulates a series of NK cell receptors that are critical for tumour cell targeting: NKp30 (binding to B7-H6 on tumour cells), DNAM-1 (binding to PVR and Nectin-2) and NKG2D (binding to MICA/B and ULBP1-6 ligands). Conversely, DOT-cell activity is negatively regulated by TIGIT and PD-1, which are induced in the tumour microenvironment. The DOT-cell protocol also upregulates chemokine receptors, like CXCR3 and CXCR4, that control their migration and infiltration into tumour lesions; and several molecules associated with tissue retention and residency, namely CD69 or CD103, alike tissue-resident memory T cells. Finally, DOT cells are very amenable to genetic engineering, and transduction with chimeric antigen receptors (CAR) specific for tumour-associated antigens (TAA) enhances their potency in vitro and in vivo.

Mechanistic studies indicated that DOT-cell recognition of haematological tumours is mediated by a combination of TCR-dependent and innate receptor–dependent pathways, highlighting the role of NK cell receptors (NKRs) expressed at high levels as result of TCR activation during in vitro DOT-cell expansion (Almeida et al., 2016) (Figure 1). Among NKRs, NKp30 and DNAM-1 were shown to be the most relevant for targeting haematological tumours (Almeida et al., 2016; Di Lorenzo et al., 2019; Mensurado et al., 2024). In particular, the NKp30 ligand, B7-H6, and the DNAM-1 ligand, CD155/PVR, acted in non-redundant and additive manners to promote immune synapse formation, cytoskeletal polarization, and perforin-mediated killing of AML cells (Mensurado et al., 2024). Genetic ablation of either ligand impaired DOT-cell cytotoxicity, and their combined ablation further decreased AML cell targeting in vitro and in vivo. Notably, while both ligands contributed to the elimination of AML cell lines, PVR expression uniquely predicted the susceptibility of primary AML samples to DOT-cell killing, positioning it as a potential biomarker of response in clinical studies (Mensurado et al., 2024). Overall, by providing broad tumour cell recognition and resistance to clonal escape, DOT cells may address key challenges posed by haematological diseases, offering a compelling foundation for their continued clinical development.

## DOT cells in solid cancers

Since Vδ1^+^ T cells are typically enriched within healthy and malignant tissues (de Vries et al., 2023; Wu et al., 2019, 2022; Rancan et al., 2023), they are logical candidates for immunotherapy of solid cancers. Although derived from peripheral blood, DOT cells acquire a broad repertoire of tissue- and tumour-homing receptors during in vitro expansion (Almeida et al., 2016) enabling efficient trafficking to solid tumours. In orthotopic xenograft models of colorectal cancer (CRC), infused DOT cells readily infiltrated tumours and inhibited tumour growth (Blanco-Domínguez et al., 2025a). Importantly, analyses using colorectal cancer (CRC) cell lines and patient-derived organoids (PDOs) demonstrated that DOT-cell cytotoxicity targets mismatch repair-deficient (dMMR) and MMR-proficient (pMMR) that are typically immune-checkpoint-blockade-resistant tumours, seemingly attributable to their NKR-mediated recognition mechanisms (Blanco-Domínguez et al., 2025a).

Additionally, several strategies proved capable of overcoming the suppressive tumour microenvironment (TME). Thus, DOT cells isolated from CRC xenografts shared with tumour-infiltrating Vδ1^+^ T cells from CRC patients elevated PD-1 and TIGIT expression levels, which resulted in impaired effector activity upon ligand engagement (Figure 1). Critically, combined PD-1/TIGIT blockade restored DOT-cell cytotoxicity and markedly enhanced tumour control in vivo (Blanco-Domínguez et al., 2025a). Together with evidence that CRC-infiltrating Vδ1^+^ T cells can respond to anti-PD-1 therapy (de Vries et al., 2023), these findings highlight that ICB could be combined with DOT cells to enhance anti-tumour activities of endogenous and infused Vδ1^+^ T cells.

Enhancing innate tumour recognition also augments DOT-cell activity. For example, the microbiota-derived short-chain fatty acid butyrate, a histone deacetylase inhibitor, increases NKG2D ligand expression on tumour cells, and butyrate supplementation boosted NKG2D-dependent DOT-cell recognition in PDOs and tumour control in CRC xenografts (Blanco-Domínguez et al., 2025a). Another epigenetic modulator, the DNA methyltransferase inhibitor decitabine, also upregulated NKG2D ligand expression and improved DOT-cell-mediated tumour control in subcutaneous lung cancer models (Weng et al., 2021). These agents also intrinsically promote γδ T cell effector function: butyrate upregulates NKG2D and decitabine upregulates DNAM-1. This stands in contrast to the inhibitory effects of decitabine on in vitro-expanded Vδ2^+^ T cells, possibly linked to their higher propensity to functional exhaustion (Niu et al., 2018). Importantly, despite the limitations of the in vivo models employed in these studies, both butyrate and decitabine produced minimal off-target toxicity (Weng et al., 2021; Blanco-Domínguez et al., 2025a), highlighting their potential for safe integration into DOT-cell-based therapeutic strategies.

Regulatory T (Treg) cells also contribute to the immune suppressive TME. We recently showed that Treg cells may inhibit DOT cells and their type 1 cytotoxic mouse Vγ1^+^ γδ T cell counterparts, by outcompeting them for IL-2, a key cytokine for anti-tumour γδ T-cell proliferation and function (Blanco-Domínguez et al., 2025b). Treg cells gain this competitive advantage through constitutive expression of CD25, the high affinity □□ chain of the IL-2R. We demonstrated that neoleukin-2/15, a synthetic IL-2/IL-15 receptor agonist that signals independently of CD25, overcomes Treg-mediated suppression and enhances DOT-cell activity in an orthotopic breast cancer xenograft model, thus suggesting therapeutic potential for future combinatorial approaches (Blanco-Domínguez et al., 2025b).

## Future perspectives

As we expect that DOT cell-based products will prove safe in the clinic (NCT05886491), the key question becomes how to improve their efficacy for the treatment of haematological or solid cancers. One immediate area of refinement lies in improving effector DOT-cell differentiation during in vitro expansion. Recent work from Lynch and colleagues showed that incorporating IL-18 and anti-CD2 stimulation while omitting IL-1β in the “DOT protocol” enhanced effector differentiation, metabolic fitness, and anti-tumour activity of Vδ1^+^ T cell-based products, resulting in improved tumour control in CRC models (Harmon et al., 2023).

Beyond culture refinement, genetic engineering markedly improves DOT-cell efficacy in pre-clinical models of AML. DOT cells have been successfully used as a vehicle for chimeric antigen receptor (CAR) expression targeting CD123 (Sánchez Martínez et al., 2022). CD123-directed CAR-DOT cells displayed enhanced cytotoxicity compared with unmodified DOT cells against AML cell lines and primary patient samples in vitro and in vivo. Importantly, CAR-DOT cells seemingly showed prolonged persistence and superior activity compared to unmodified DOT cells upon tumour rechallenge in xenograft models, providing proof-of-concept for DOT cells as a *bona fide* allogeneic CAR-T cell platform (Sánchez Martínez et al., 2022).

Rapid approval of combinatorial strategies can be frustratingly impeded by regulatory processes, but nonetheless DOT-cell therapy could conceivably permit dose-reduction of standard-of-care ICB (anti-PD-1) thereby reducing widespread toxicities. Similarly, the data support possible synergies with anti-TIGIT or neoleukin-2/15. Owing to DOT-cell dependence on IL-15 or IL-2 signalling (Sánchez Martínez et al., 2022; Blanco-Domínguez et al., 2025a; b), its endogenous availability or exogenous triggering will be important.

Finally, given the pivotal role of TCR signalling in driving DOT-cell proliferation and type 1 effector functions, we believe that TCR engagement, incorporated in bispecific or trispecific modalities, can enhance DOT-cell activity after infusion, and particularly in the TME, where it is unclear if natural Vδ1^+^ TCR ligands are plentiful. In fact, Vδ1^+^ T-cell engagers alone may be important therapeutic agents, if they are able to drive sufficient activation and expansion of endogenous Vδ1^+^ T cells to deliver unrelenting force. The efficacy (and safety) of this approach should be compared to (engineered) DOT-cell-based adoptive therapy to find the best avenue to translate the unique properties of Vδ1^+^ T cells into treating more patients and more cancer types.
